# 2354. Factors associated with COVID-19 booster vaccine hesitancy: a nation-wide, cross-sectional survey in Japan

**DOI:** 10.1093/ofid/ofad500.1975

**Published:** 2023-11-27

**Authors:** Hitoshi Honda, Akane Takamatsu, Toshiki Miwa, Yasuharu Tokuda

**Affiliations:** Fujita Health University School of Medicine , Toyoake, Aichi, Japan; Tokyo Metropolitan Tama Medical Center, Fuchu, Tokyo, Japan; University of Tokyo Hospital, Tokyo, Tokyo, Japan; Muribushi Project for Teaching Hospitals, Urasoe, Okinawa, Japan

## Abstract

**Background:**

COVID-19 vaccine hesitancy and/or fatigue is increasing as the pandemic enters the endemic phase. The present study aimed to explore current perceptions about COVID-19 booster vaccination among the Japanese public.

**Methods:**

This cross-sectional study used data from the Japan COVID-19 and Society Internet Survey conducted in September 2021 and September 2022. The public’s perceptions of COVID-19 vaccination and factors associated with COVID-19 booster vaccine hesitancy were analyzed using multivariable logistic regression analysis.

**Results:**

In total, 56,735 respondents were included. In the 2022 survey, 74.1% of the respondents (21,216/28,617) completed the primary series of vaccination with booster doses. Factors independently associated with booster vaccine hesitancy were young age (range: 18-29 years; adjusted odds ratio [aOR]: 6.56; 95% confidence interval [CI]: 5.07-8.47), history of COVID-19 (aOR: 1.82; 95% CI: 1.59-2.08), distrust of the Japanese government’s COVID-19 prevention measures (aOR: 1.55; 95% CI: 1.15-2.10), lack of confidence in COVID-19 vaccine efficacy (aOR: 1.30; 95% CI: 1.02-1.65), lack of confidence in COVID-19 vaccine safety (aOR: 1.62; 95% CI: 1.35-1.94), low reliance on the COVID-19 vaccine (aOR: 1.92; 95% CI: 1.35-2.73), and belief in COVID-19 conspiracy theories (aOR: 1.77; 95% CI: 1.17-2.67).

Respondents’ selection

**Figure:**
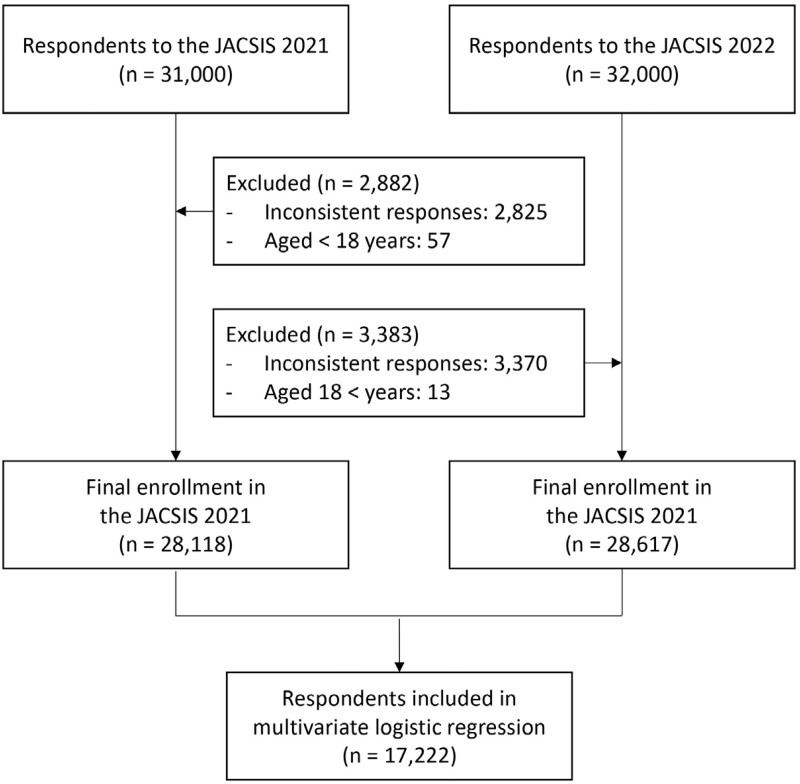

Multivariate analysis of factors associated with COVID-19 booster vaccination hesitancy (n = 17,222)

**Figure:**
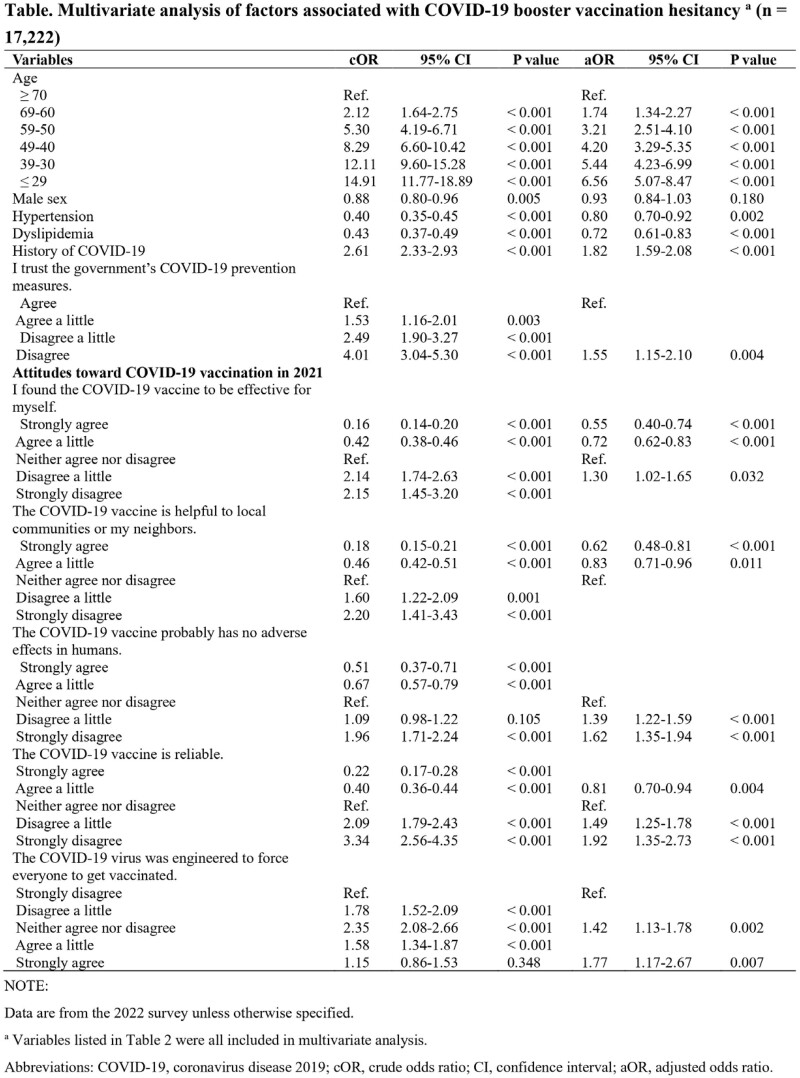

**Conclusion:**

Providing clear and trustworthy information is critically important to promoting COVID-19 booster vaccination. Policymakers should therefore develop consistent and transparent communication strategies and the ability to respond promptly and flexibly to mitigate the negative impact of COVID-19 on the public while preparing for the next pandemic.

**Disclosures:**

**Hitoshi Honda, MD**, Moderna: Honoraria

